# Methyl 7-oxo-12-propyl­amino-13-nitro­deisopropyl­dehydro­abietate

**DOI:** 10.1107/S1600536810032824

**Published:** 2010-09-25

**Authors:** Kai Wang, Ye Zhang, Xiang-Hui Yi, Yong Zhang, Ying-Ming Pan

**Affiliations:** a College of Chemistry and Chemical Engineering, Guangxi Normal University, Guilin 541004, People’s Republic of China; b Department of Chemistry and Engineering Technology, Guilin Normal College, Guilin 541004, People’s Republic of China; c College of Chemistry and Chemical Engineering, Suzhou University, Suzhou 215006, People’s Republic of China

## Abstract

In the title compound, C_21_H_28_N_2_O_5_ (systematic name: methyl 1,4a-dimethyl-7-nitro-9-oxo-6-propyl­amino-1,2,3,4,4a,9,10,10a-octa­hydro­phenanthrene-1-carboxyl­ate) the cyclo­hexane ring (*A*) and the central cyclo­hexene ring (*B*) exist at a *trans* ring junction, with the two methyl groups in the axial positions of the six-membered rings. Ring *A* has a chair conformation and ring *B* a half-chair conformation. An intra­molecular N—H⋯O hydrogen bond occurs. The crystal structure is stabilized by inter­molecular C—H⋯O and N—H⋯O inter­actions.

## Related literature

For inhibition of viruses by resin acid derivatives, see: Fonseca *et al.* (2004 ▶); Gigante *et al.* (2003 ▶). For related structures, see: Hamodrakas *et al.* (1978 ▶); Silvestre *et al.* (1998 ▶). 
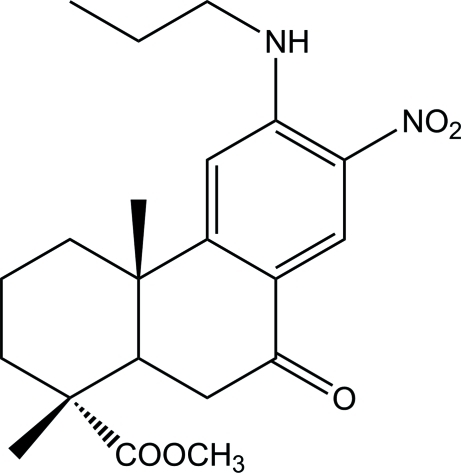


## Experimental

### 

#### Crystal data

C_21_H_28_N_2_O_5_
*M* *_r_* = 388.45
Orthorhombic, 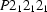

*a* = 8.2915 (15) Å
*b* = 11.344 (2) Å
*c* = 20.288 (4) Å
*V* = 1908.3 (6) Å^3^
*Z* = 4
Mo *K*α radiation
μ = 0.10 mm^−1^
*T* = 223 K
0.40 × 0.18 × 0.14 mm

#### Data collection

Rigaku Saturn diffractometer
Absorption correction: multi-scan (Jacobson, 1998 ▶) *T*_min_ = 0.956, *T*_max_ = 0.987
9263 measured reflections
2492 independent reflections
2249 reflections with *I* > 2σ(*I*)
*R*_int_ = 0.047

#### Refinement

*R*[*F* ^2^ > 2σ(*F* ^2^)] = 0.061
*wR*(*F* ^2^) = 0.122
*S* = 1.19
2492 reflections
262 parameters
H atoms treated by a mixture of independent and constrained refinement
Δρ_max_ = 0.16 e Å^−3^
Δρ_min_ = −0.20 e Å^−3^

### 

Data collection: *CrystalClear* (Rigaku, 1999 ▶); cell refinement: *CrystalClear*; data reduction: *CrystalStructure* (Rigaku/MSC, 2000 ▶); program(s) used to solve structure: *SHELXS97* (Sheldrick, 2008 ▶); program(s) used to refine structure: *SHELXL97* (Sheldrick, 2008 ▶); molecular graphics: *SHELXTL* (Sheldrick, 2008 ▶); software used to prepare material for publication: *SHELXTL*.

## Supplementary Material

Crystal structure: contains datablocks global, I. DOI: 10.1107/S1600536810032824/ng5010sup1.cif

Structure factors: contains datablocks I. DOI: 10.1107/S1600536810032824/ng5010Isup2.hkl

Additional supplementary materials:  crystallographic information; 3D view; checkCIF report

## Figures and Tables

**Table 1:** Hydrogen-bond geometry (Å, °)

*D*—H⋯*A*	*D*—H	H⋯*A*	*D*⋯*A*	*D*—H⋯*A*
N1—H1*A*⋯O3	0.93 (4)	1.94 (4)	2.654 (4)	133 (3)
C8—H8*B*⋯O3^i^	0.98	2.55	3.454 (4)	154
C15—H15*A*⋯O1^ii^	0.98	2.40	3.344 (4)	161

Symmetry codes: (i) 
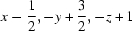
; (ii) 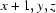.

**Fig. 1.**
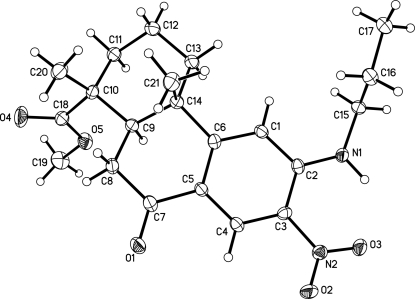
The structure of the title compound showing 50% probability displacement ellipsoids and the atom labelling scheme. H atoms are represented by small spheres of arbitrary radius.

**Fig. 2.**
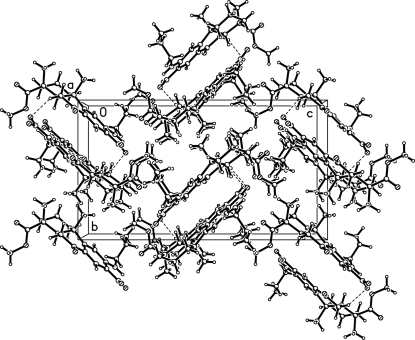
Packing diagram with H bonds indicated by dashed lines.

## supplementary crystallographic information

### Comment

As the main components of rosin, abietic acid and dehydroabietic acid are tricyclic diterpene carboxylic acids. It have been demonstrated that resin acid derivatives exhibit inhibition activity against viruses by recent works (Gigante *et al.*, 2003; Fonseca *et al.*, 2004), which prompted us to synthesis of the title compound. In the cation of the title compounds (Fig.1), rings A (atoms C9—C14) and rings B (atoms C5—C10) demonstrate a *trans* ring junction with the torsion angles showing classical chair and halfchair conformations for rings A and B, respectively. There are two methyl groups in the axial positions of the six-membered rings and the overall geometric parameters of the title compound are comparable to those of 12-acetyl-dehydroabietate (Silvestre, *et al.*, 1998) and methyl dehydroabietate (Hamodrakas, *et al.*, 1978), apart from the substituted nitro group and propylamino at the benzene ring.

It should be noted that there are weak intermolecular C—H···O and N—H···O hydrogen bonds in the packing view, which link the molecules into a one-dimensional chain to stabilize the structure (Fig. 2).

The absolute configuration of the title compound could not determined from anomalous scattering effects because none heavier atoms than Si are present. However, NMR studies of analgous compounds suggest that the configuration is retained through the course of the reaction.Therefore, the absolute configuration of the title compound is assumed from the known absolute configuration of methyl dehydroabietate (Hamodrakas, *et al.*, 1978).

### Experimental

Methyl 7-oxo-12-bromo-13-nitro-deisopropyldehydroabietate (2.5 mmol), potassium carbonate (1.0 mmol), cuprous chloride (2.0 mmol) were added to 15 ml DMF. After stirring for 10 min, n-propylamine (2.5 mmol) was added drop-wise. The resultant solution was refluxed for 4 h, and then plenty of ice water was added, a lot of orange-yellow solid was precipitated, filtered, washed with water, and then dried. Upon recrystallization from ethanol, pale orange crystls were obtained (Yield 78.9%, m.p. 455–456 k).

### Refinement

H atoms bound to C atoms were positioned geometrically and included in the refinement in the riding-model approximation [*d*(C—H) = 0.95 and 0.99 Å for aromatic and CH_2_ groups, respectively, and with *U*_iso_(H) = 1.2*U*_eq_ (C) for all others. 1443 Friedel pairs were merged.

### Crystal data

**Table:**

C_21_H_28_N_2_O_5_	*F*(000) = 832
*M**_r_* = 388.45	*D*_x_ = 1.352 Mg m^−^^3^
Orthorhombic, *P*2_1_2_1_2_1_	Mo *K*α radiation, λ = 0.71075 Å
Hall symbol: P 2ac 2ab	Cell parameters from 6184 reflections
*a* = 8.2915 (15) Å	θ = 3.0–27.5°
*b* = 11.344 (2) Å	µ = 0.10 mm^−^^1^
*c* = 20.288 (4) Å	*T* = 223 K
*V* = 1908.3 (6) Å^3^	Block, yellow
*Z* = 4	0.40 × 0.18 × 0.14 mm

### Data collection

**Table:**

Rigaku Saturn diffractometer	2492 independent reflections
Radiation source: fine-focus sealed tube	2249 reflections with *I* > 2σ(*I*)
graphite	*R*_int_ = 0.047
Detector resolution: 14.63 pixels mm^-1^	θ_max_ = 27.5°, θ_min_ = 3.0°
ω scans	*h* = −10→10
Absorption correction: multi-scan (Jacobson, 1998)	*k* = −13→14
*T*_min_ = 0.956, *T*_max_ = 0.987	*l* = −26→18
9263 measured reflections	

### Refinement

**Table:**

Refinement on *F*^2^	Primary atom site location: structure-invariant direct methods
Least-squares matrix: full	Secondary atom site location: difference Fourier map
*R*[*F*^2^ > 2σ(*F*^2^)] = 0.061	Hydrogen site location: inferred from neighbouring sites
*wR*(*F*^2^) = 0.122	H atoms treated by a mixture of independent and constrained refinement
*S* = 1.19	*w* = 1/[σ^2^(*F*_o_^2^) + (0.040*P*)^2^ + 0.3656*P*] where *P* = (*F*_o_^2^ + 2*F*_c_^2^)/3
2492 reflections	(Δ/σ)_max_ < 0.001
262 parameters	Δρ_max_ = 0.16 e Å^−^^3^
0 restraints	Δρ_min_ = −0.20 e Å^−^^3^

### Special details

**Table:**

Geometry. All e.s.d.'s (except the e.s.d. in the dihedral angle between two l.s. planes) are estimated using the full covariance matrix. The cell e.s.d.'s are taken into account individually in the estimation of e.s.d.'s in distances, angles and torsion angles; correlations between e.s.d.'s in cell parameters are only used when they are defined by crystal symmetry. An approximate (isotropic) treatment of cell e.s.d.'s is used for estimating e.s.d.'s involving l.s. planes.
Refinement. Refinement of *F*^2^ against ALL reflections. The weighted *R*-factor *wR* and goodness of fit *S* are based on *F*^2^, conventional *R*-factors *R* are based on *F*, with *F* set to zero for negative *F*^2^. The threshold expression of *F*^2^ > σ(*F*^2^) is used only for calculating *R*-factors(gt) *etc*. and is not relevant to the choice of reflections for refinement. *R*-factors based on *F*^2^ are statistically about twice as large as those based on *F*, and *R*- factors based on ALL data will be even larger.

### Fractional atomic coordinates and isotropic or equivalent isotropic displacement parameters (Å^2^)

**Table:**

	*x*	*y*	*z*	*U*_iso_*/*U*_eq_	
O1	−0.0800 (3)	0.5805 (2)	0.50671 (13)	0.0413 (7)	
O2	0.1082 (3)	0.8201 (2)	0.32927 (14)	0.0428 (7)	
O3	0.3574 (3)	0.8723 (2)	0.31899 (13)	0.0374 (6)	
O4	0.1294 (4)	0.3814 (3)	0.75394 (15)	0.0594 (9)	
O5	0.2387 (3)	0.5514 (2)	0.72342 (13)	0.0393 (6)	
N1	0.5809 (3)	0.7320 (3)	0.37018 (15)	0.0301 (7)	
H1A	0.552 (4)	0.784 (3)	0.3368 (19)	0.034 (10)*	
N2	0.2510 (3)	0.8110 (3)	0.34543 (14)	0.0296 (6)	
C1	0.4799 (4)	0.5968 (3)	0.45202 (16)	0.0273 (7)	
H1B	0.5847	0.5672	0.4583	0.033*	
C2	0.4562 (4)	0.6880 (3)	0.40463 (16)	0.0261 (7)	
C3	0.2948 (4)	0.7264 (3)	0.39580 (16)	0.0264 (7)	
C4	0.1710 (4)	0.6826 (3)	0.43411 (16)	0.0272 (7)	
H4	0.0662	0.7126	0.4284	0.033*	
C5	0.1976 (4)	0.5958 (3)	0.48066 (16)	0.0259 (7)	
C6	0.3556 (4)	0.5500 (3)	0.48907 (15)	0.0249 (7)	
C7	0.0594 (4)	0.5512 (3)	0.51883 (17)	0.0306 (8)	
C8	0.0933 (4)	0.4657 (3)	0.57383 (17)	0.0291 (7)	
H8A	0.0626	0.3863	0.5595	0.035*	
H8B	0.0262	0.4863	0.6119	0.035*	
C9	0.2698 (4)	0.4647 (3)	0.59499 (15)	0.0255 (7)	
H9	0.2930	0.5455	0.6106	0.031*	
C10	0.2965 (4)	0.3826 (3)	0.65599 (17)	0.0285 (7)	
C11	0.4779 (4)	0.3807 (3)	0.67347 (18)	0.0336 (8)	
H11A	0.5099	0.4585	0.6899	0.040*	
H11B	0.4962	0.3233	0.7088	0.040*	
C12	0.5822 (5)	0.3488 (4)	0.61471 (19)	0.0382 (9)	
H12A	0.5536	0.2697	0.5992	0.046*	
H12B	0.6958	0.3477	0.6281	0.046*	
C13	0.5592 (4)	0.4378 (3)	0.55853 (18)	0.0339 (8)	
H13A	0.6285	0.4154	0.5214	0.041*	
H13B	0.5933	0.5160	0.5737	0.041*	
C14	0.3833 (4)	0.4444 (3)	0.53470 (16)	0.0264 (7)	
C15	0.7445 (4)	0.6817 (3)	0.36949 (17)	0.0294 (7)	
H15A	0.7771	0.6639	0.4148	0.035*	
H15B	0.8197	0.7406	0.3520	0.035*	
C16	0.7571 (4)	0.5698 (3)	0.32818 (18)	0.0346 (8)	
H16A	0.7319	0.5881	0.2821	0.042*	
H16B	0.6781	0.5120	0.3439	0.042*	
C17	0.9260 (4)	0.5170 (3)	0.33244 (19)	0.0364 (9)	
H17A	1.0049	0.5762	0.3202	0.055*	
H17B	0.9340	0.4504	0.3026	0.055*	
H17C	0.9465	0.4909	0.3772	0.055*	
C18	0.2105 (4)	0.4364 (3)	0.71584 (18)	0.0326 (8)	
C19	0.1741 (5)	0.6042 (4)	0.7827 (2)	0.0500 (11)	
H19A	0.0574	0.5989	0.7821	0.075*	
H19B	0.2061	0.6864	0.7848	0.075*	
H19C	0.2156	0.5627	0.8210	0.075*	
C20	0.2305 (5)	0.2577 (3)	0.6477 (2)	0.0412 (9)	
H20A	0.2449	0.2143	0.6885	0.062*	
H20B	0.2882	0.2182	0.6125	0.062*	
H20C	0.1167	0.2613	0.6369	0.062*	
C21	0.3452 (5)	0.3342 (3)	0.49327 (18)	0.0376 (9)	
H21A	0.4110	0.3344	0.4538	0.056*	
H21B	0.2321	0.3347	0.4811	0.056*	
H21C	0.3684	0.2640	0.5189	0.056*	

### Atomic displacement parameters (Å^2^)

**Table:**

	*U*^11^	*U*^22^	*U*^33^	*U*^12^	*U*^13^	*U*^23^
O1	0.0236 (12)	0.0637 (19)	0.0365 (15)	0.0019 (13)	0.0007 (11)	0.0130 (14)
O2	0.0259 (13)	0.0531 (16)	0.0492 (17)	0.0044 (13)	−0.0076 (13)	0.0179 (14)
O3	0.0346 (13)	0.0395 (14)	0.0382 (15)	−0.0046 (12)	0.0018 (12)	0.0126 (12)
O4	0.077 (2)	0.0477 (17)	0.053 (2)	0.0017 (17)	0.0362 (18)	0.0111 (15)
O5	0.0461 (15)	0.0413 (14)	0.0305 (14)	0.0002 (13)	0.0097 (12)	−0.0060 (12)
N1	0.0234 (14)	0.0372 (17)	0.0299 (16)	−0.0009 (13)	0.0009 (12)	0.0100 (13)
N2	0.0298 (15)	0.0315 (15)	0.0275 (14)	0.0017 (14)	−0.0013 (13)	0.0004 (13)
C1	0.0216 (14)	0.0353 (19)	0.0248 (17)	0.0028 (14)	0.0011 (13)	0.0005 (15)
C2	0.0239 (16)	0.0304 (18)	0.0239 (17)	−0.0055 (15)	0.0017 (13)	−0.0018 (15)
C3	0.0275 (16)	0.0288 (17)	0.0228 (16)	0.0026 (15)	−0.0052 (13)	0.0015 (14)
C4	0.0210 (15)	0.0338 (18)	0.0268 (17)	−0.0007 (15)	0.0008 (13)	−0.0020 (14)
C5	0.0214 (14)	0.0314 (17)	0.0250 (17)	0.0004 (14)	0.0019 (12)	−0.0001 (14)
C6	0.0251 (15)	0.0306 (17)	0.0191 (15)	−0.0040 (14)	−0.0012 (13)	−0.0021 (13)
C7	0.0255 (16)	0.039 (2)	0.0271 (18)	−0.0021 (16)	0.0007 (14)	−0.0005 (16)
C8	0.0255 (16)	0.0344 (19)	0.0274 (18)	−0.0031 (15)	0.0029 (13)	0.0044 (15)
C9	0.0261 (17)	0.0260 (17)	0.0245 (16)	0.0017 (15)	0.0013 (13)	−0.0003 (13)
C10	0.0323 (17)	0.0271 (17)	0.0261 (17)	0.0002 (15)	0.0035 (14)	0.0041 (14)
C11	0.0343 (18)	0.039 (2)	0.0271 (18)	0.0047 (16)	0.0000 (15)	0.0068 (16)
C12	0.0332 (19)	0.046 (2)	0.036 (2)	0.0116 (18)	0.0032 (17)	0.0074 (17)
C13	0.0305 (17)	0.042 (2)	0.0290 (19)	0.0076 (17)	0.0032 (14)	0.0060 (16)
C14	0.0274 (16)	0.0258 (17)	0.0259 (17)	0.0019 (15)	0.0030 (13)	−0.0010 (14)
C15	0.0202 (15)	0.0356 (19)	0.0326 (18)	0.0007 (16)	−0.0007 (14)	0.0027 (15)
C16	0.0302 (17)	0.040 (2)	0.0332 (19)	−0.0007 (17)	0.0017 (15)	−0.0035 (16)
C17	0.0303 (17)	0.041 (2)	0.038 (2)	0.0018 (17)	0.0047 (16)	−0.0022 (17)
C18	0.0332 (18)	0.0372 (19)	0.0272 (18)	0.0019 (16)	0.0003 (14)	0.0058 (16)
C19	0.061 (3)	0.059 (3)	0.030 (2)	0.010 (2)	0.007 (2)	−0.013 (2)
C20	0.051 (2)	0.0318 (19)	0.041 (2)	0.0020 (19)	0.0046 (19)	0.0052 (17)
C21	0.051 (2)	0.035 (2)	0.0275 (19)	−0.0011 (18)	0.0040 (17)	−0.0043 (16)

### Geometric parameters (Å, °)

**Table:**

O1—C7	1.227 (4)	C10—C11	1.545 (5)
O2—N2	1.233 (3)	C11—C12	1.517 (5)
O3—N2	1.245 (3)	C11—H11A	0.9800
O4—C18	1.200 (4)	C11—H11B	0.9800
O5—C18	1.334 (4)	C12—C13	1.535 (5)
O5—C19	1.447 (4)	C12—H12A	0.9800
N1—C2	1.344 (4)	C12—H12B	0.9800
N1—C15	1.472 (4)	C13—C14	1.539 (5)
N1—H1A	0.93 (4)	C13—H13A	0.9800
N2—C3	1.448 (4)	C13—H13B	0.9800
C1—C6	1.382 (4)	C14—C21	1.540 (5)
C1—C2	1.426 (5)	C15—C16	1.525 (5)
C1—H1B	0.9400	C15—H15A	0.9800
C2—C3	1.418 (4)	C15—H15B	0.9800
C3—C4	1.380 (5)	C16—C17	1.525 (5)
C4—C5	1.383 (5)	C16—H16A	0.9800
C4—H4	0.9400	C16—H16B	0.9800
C5—C6	1.419 (4)	C17—H17A	0.9700
C5—C7	1.472 (4)	C17—H17B	0.9700
C6—C14	1.531 (5)	C17—H17C	0.9700
C7—C8	1.505 (5)	C19—H19A	0.9700
C8—C9	1.525 (4)	C19—H19B	0.9700
C8—H8A	0.9800	C19—H19C	0.9700
C8—H8B	0.9800	C20—H20A	0.9700
C9—C14	1.560 (4)	C20—H20B	0.9700
C9—C10	1.564 (5)	C20—H20C	0.9700
C9—H9	0.9900	C21—H21A	0.9700
C10—C20	1.529 (5)	C21—H21B	0.9700
C10—C18	1.535 (5)	C21—H21C	0.9700
			
C18—O5—C19	115.8 (3)	C11—C12—H12B	109.5
C2—N1—C15	124.8 (3)	C13—C12—H12B	109.5
C2—N1—H1A	115 (2)	H12A—C12—H12B	108.1
C15—N1—H1A	118 (2)	C12—C13—C14	112.5 (3)
O2—N2—O3	121.3 (3)	C12—C13—H13A	109.1
O2—N2—C3	119.0 (3)	C14—C13—H13A	109.1
O3—N2—C3	119.7 (3)	C12—C13—H13B	109.1
C6—C1—C2	122.8 (3)	C14—C13—H13B	109.1
C6—C1—H1B	118.6	H13A—C13—H13B	107.8
C2—C1—H1B	118.6	C6—C14—C13	111.8 (3)
N1—C2—C3	123.1 (3)	C6—C14—C21	105.9 (3)
N1—C2—C1	120.9 (3)	C13—C14—C21	109.0 (3)
C3—C2—C1	116.0 (3)	C6—C14—C9	105.6 (3)
C4—C3—C2	121.4 (3)	C13—C14—C9	109.4 (3)
C4—C3—N2	116.7 (3)	C21—C14—C9	115.1 (3)
C2—C3—N2	122.0 (3)	N1—C15—C16	113.0 (3)
C3—C4—C5	121.5 (3)	N1—C15—H15A	109.0
C3—C4—H4	119.3	C16—C15—H15A	109.0
C5—C4—H4	119.3	N1—C15—H15B	109.0
C4—C5—C6	119.3 (3)	C16—C15—H15B	109.0
C4—C5—C7	118.7 (3)	H15A—C15—H15B	107.8
C6—C5—C7	122.0 (3)	C15—C16—C17	111.0 (3)
C1—C6—C5	118.9 (3)	C15—C16—H16A	109.4
C1—C6—C14	121.1 (3)	C17—C16—H16A	109.4
C5—C6—C14	119.8 (3)	C15—C16—H16B	109.4
O1—C7—C5	122.3 (3)	C17—C16—H16B	109.4
O1—C7—C8	119.9 (3)	H16A—C16—H16B	108.0
C5—C7—C8	117.8 (3)	C16—C17—H17A	109.5
C7—C8—C9	113.1 (3)	C16—C17—H17B	109.5
C7—C8—H8A	109.0	H17A—C17—H17B	109.5
C9—C8—H8A	109.0	C16—C17—H17C	109.5
C7—C8—H8B	109.0	H17A—C17—H17C	109.5
C9—C8—H8B	109.0	H17B—C17—H17C	109.5
H8A—C8—H8B	107.8	O4—C18—O5	122.2 (4)
C8—C9—C14	111.1 (3)	O4—C18—C10	124.3 (3)
C8—C9—C10	111.3 (3)	O5—C18—C10	113.5 (3)
C14—C9—C10	116.6 (3)	O5—C19—H19A	109.5
C8—C9—H9	105.7	O5—C19—H19B	109.5
C14—C9—H9	105.7	H19A—C19—H19B	109.5
C10—C9—H9	105.7	O5—C19—H19C	109.5
C20—C10—C18	106.8 (3)	H19A—C19—H19C	109.5
C20—C10—C11	111.1 (3)	H19B—C19—H19C	109.5
C18—C10—C11	106.1 (3)	C10—C20—H20A	109.5
C20—C10—C9	114.5 (3)	C10—C20—H20B	109.5
C18—C10—C9	108.9 (3)	H20A—C20—H20B	109.5
C11—C10—C9	109.2 (3)	C10—C20—H20C	109.5
C12—C11—C10	112.2 (3)	H20A—C20—H20C	109.5
C12—C11—H11A	109.2	H20B—C20—H20C	109.5
C10—C11—H11A	109.2	C14—C21—H21A	109.5
C12—C11—H11B	109.2	C14—C21—H21B	109.5
C10—C11—H11B	109.2	H21A—C21—H21B	109.5
H11A—C11—H11B	107.9	C14—C21—H21C	109.5
C11—C12—C13	110.8 (3)	H21A—C21—H21C	109.5
C11—C12—H12A	109.5	H21B—C21—H21C	109.5
C13—C12—H12A	109.5		
			
C15—N1—C2—C3	−169.2 (3)	C14—C9—C10—C18	164.2 (3)
C15—N1—C2—C1	9.6 (5)	C8—C9—C10—C11	177.6 (3)
C6—C1—C2—N1	179.2 (3)	C14—C9—C10—C11	48.8 (4)
C6—C1—C2—C3	−2.0 (5)	C20—C10—C11—C12	73.9 (4)
N1—C2—C3—C4	−177.2 (3)	C18—C10—C11—C12	−170.4 (3)
C1—C2—C3—C4	4.0 (5)	C9—C10—C11—C12	−53.2 (4)
N1—C2—C3—N2	4.2 (5)	C10—C11—C12—C13	59.8 (4)
C1—C2—C3—N2	−174.6 (3)	C11—C12—C13—C14	−59.2 (4)
O2—N2—C3—C4	−15.9 (5)	C1—C6—C14—C13	26.8 (4)
O3—N2—C3—C4	164.6 (3)	C5—C6—C14—C13	−157.9 (3)
O2—N2—C3—C2	162.7 (3)	C1—C6—C14—C21	−91.8 (4)
O3—N2—C3—C2	−16.7 (5)	C5—C6—C14—C21	83.5 (4)
C2—C3—C4—C5	−3.0 (5)	C1—C6—C14—C9	145.7 (3)
N2—C3—C4—C5	175.7 (3)	C5—C6—C14—C9	−39.0 (4)
C3—C4—C5—C6	−0.3 (5)	C12—C13—C14—C6	168.4 (3)
C3—C4—C5—C7	−178.7 (3)	C12—C13—C14—C21	−74.8 (4)
C2—C1—C6—C5	−1.1 (5)	C12—C13—C14—C9	51.8 (4)
C2—C1—C6—C14	174.2 (3)	C8—C9—C14—C6	62.4 (3)
C4—C5—C6—C1	2.3 (5)	C10—C9—C14—C6	−168.7 (3)
C7—C5—C6—C1	−179.4 (3)	C8—C9—C14—C13	−177.2 (3)
C4—C5—C6—C14	−173.1 (3)	C10—C9—C14—C13	−48.3 (4)
C7—C5—C6—C14	5.2 (5)	C8—C9—C14—C21	−54.0 (4)
C4—C5—C7—O1	6.8 (5)	C10—C9—C14—C21	74.9 (4)
C6—C5—C7—O1	−171.5 (3)	C2—N1—C15—C16	75.2 (4)
C4—C5—C7—C8	−174.0 (3)	N1—C15—C16—C17	−176.5 (3)
C6—C5—C7—C8	7.7 (5)	C19—O5—C18—O4	3.8 (5)
O1—C7—C8—C9	−164.0 (3)	C19—O5—C18—C10	−173.9 (3)
C5—C7—C8—C9	16.8 (4)	C20—C10—C18—O4	11.4 (5)
C7—C8—C9—C14	−53.1 (4)	C11—C10—C18—O4	−107.1 (4)
C7—C8—C9—C10	175.2 (3)	C9—C10—C18—O4	135.5 (4)
C8—C9—C10—C20	52.4 (4)	C20—C10—C18—O5	−170.8 (3)
C14—C9—C10—C20	−76.4 (4)	C11—C10—C18—O5	70.6 (4)
C8—C9—C10—C18	−67.0 (3)	C9—C10—C18—O5	−46.8 (4)

### Hydrogen-bond geometry (Å, °)

**Table:**

*D*—H···*A*	*D*—H	H···*A*	*D*···*A*	*D*—H···*A*
N1—H1A···O3	0.93 (4)	1.94 (4)	2.654 (4)	133 (3)
C8—H8B···O3^i^	0.98	2.55	3.454 (4)	154
C15—H15A···O1^ii^	0.98	2.40	3.344 (4)	161

Symmetry codes: (i) *x*−1/2, −*y*+3/2, −*z*+1; (ii) *x*+1, *y*, *z*.
